# Coordinated Regulation of Axonal Microtubule Organization and Transport by *Drosophila* Neurexin and BMP Pathway

**DOI:** 10.1038/s41598-018-35618-7

**Published:** 2018-11-26

**Authors:** Swati Banerjee, Maeveen Riordan

**Affiliations:** 1 Department of Cellular and Integrative Physiology, Long School of Medicine, University of Texas Health, 7703 Floyd Curl Drive, San Antonio, TX 78229 USA; 2 Present Address: University of Colorado School of Medicine, 12631 E. 17th Avenue B177, Aurora, CO 80045 USA

## Abstract

Neurexins are well known trans-synaptic cell adhesion molecules that are required for proper synaptic development and function across species. Beyond synapse organization and function, little is known about other roles Neurexins might have in the nervous system. Here we report novel phenotypic consequences of mutations in *Drosophila neurexin* (*dnrx*), which alters axonal microtubule organization and transport. We show that *dnrx* mutants display phenotypic similarities with the BMP receptor *wishful thinking* (*wit*) and one of the downstream effectors, *futsch*, which is a known regulator of microtubule organization and stability. *dnrx* has genetic interactions with *wit* and *futsch*. Loss of Dnrx also results in reduced levels of other downstream effectors of BMP signaling, phosphorylated-Mad and Trio. Interestingly, postsynaptic overexpression of the BMP ligand, Glass bottom boat, in *dnrx* mutants partially rescues the axonal transport defects but not the synapse undergrowth at the neuromuscular junctions. These data suggest that Dnrx and BMP signaling are involved in many diverse functions and that regulation of axonal MT organization and transport might be distinct from regulation of synaptic growth in *dnrx* mutants. Together, our work uncovers a novel function of *Drosophila* Neurexin and may provide insights into functions of Neurexins in vertebrates.

## Introduction

The synaptic cell adhesion molecule Neurexin (Nrx) is involved in the development and function of synapses across species^1–8^. Elucidating the function of Nrx is important because it is linked to many human neurodevelopmental and psychiatric disorders^4,7^. In *Drosophila*, Neurexin (Dnrx) has a critical role in the assembly and maintenance of glutamatergic neuromuscular junction (NMJ) synapses. Lack of Dnrx shows a significant reduction in synaptic bouton growth at the NMJ^3,8,9^. Our recent studies show that *dnrx* and its binding partner *neuroligin (dnlg*)^10–12^ interact with the Bone Morphogenetic Protein (BMP) receptor, *wishful thinking (wit)*, to coordinate synaptic growth and cytoarchitecture at the NMJs^8^.

The BMP pathway is a well established signaling pathway needed for the regulation of synapse assembly, maintenance, and function^13–16^. In *Drosophila*, the BMP pathway has the postsynaptic muscle-derived ligand, Glass bottom boat (Gbb) that binds to the receptors Wishful thinking (Wit), Thickveins (Tkv), and Saxophone (Sax) at the NMJ and activates a cascade of downstream effectors. One of these effectors is the phosphorylated-mothers against decapentaplegic (pMad) in neurons. pMad and the co-Smad, Medea, translocate to the nucleus and initiates transcription of downstream targets^17^. Besides NMJ growth, recent studies have shown that the BMP pathway is required for axonal microtubule (MT) stability, synaptic strength and normal axonal transport^17–20^. Axonal transport involves long-range bidirectional transport of specialized cargo, such as the components of synaptic vesicles, active zones, and organelles from soma to the nerve terminals^21,22^, and this process is powered by axonal MTs. Disruption of the delicate axonal MT organization and transport machinery has been associated with several human neurodegenerative disorders, including Alzheimer’s, Amyotrophic Lateral Sclerosis, Huntington’s and Parkinson’s diseases^23,24^. While trafficking of Neurexins at synaptic terminals has been studied^25^, not much is known about the role of Neurexins in trafficking of synaptic cargoes or assembly of MTs along the axons.

Here we report that loss of Dnrx causes axonal MT disorganization and disruption in transport that resembles phenotypes observed in *wit* mutants. *dnrx* displays genetic interactions with *wit. dnrx* also shows phenotypic similarities and genetic interactions with *futsch. futsch* is the *Drosophila* homolog of mammalian microtubule associated protein (MAP1B)^26,27^ and one of the downstream effectors of BMP pathway that regulates MT organization and stability^18,20^. Absence of Dnrx affects levels of the downstream effectors of the BMP pathway, pMad and Trio in motor neurons. Interestingly, postsynaptic overexpression of Gbb in *dnrx* mutants partially ameliorates axonal transport defects but not the NMJ undergrowth phenotype of *dnrx* mutants. Together, these data suggest that Dnrx functions with components of the BMP signaling pathway to ensure proper MT organization and axonal transport which is crucial for proper neuronal function.

## Results

### Loss and Gain of Dnrx Affects Axonal Microtubule Organization and Transport

**Figure 1:**
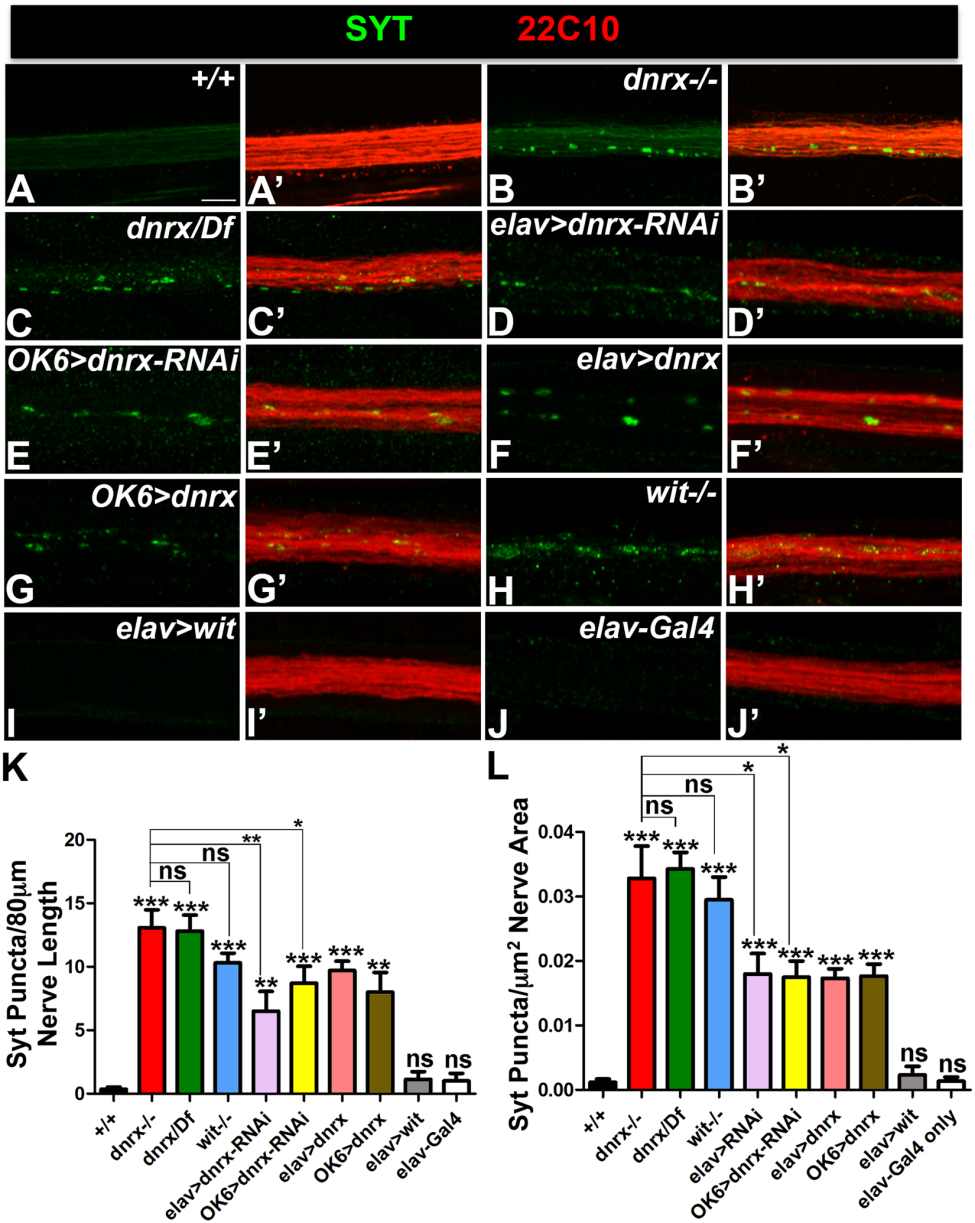
*dnrx* mutants show defects in axonal microtubule cytoskeleton and transport. (**A**–**J’**) Confocal images of 3^rd^ instar larval nerve fibers labeled with antibodies against Syt (green) and Futsch (red) in indicated genotypes. (**K**,**L**) Quantification of Syt puncta per 80 μm nerve length (**K**) and per μm^2^ nerve area of 3^rd^ instar larval segmental nerve 5–8 passing through abdominal segments A4–7 of specified genotypes. n = 15 animals. Error bars represent mean ± SEM (***p < 0.001, **p ≤ 0.01, *p ≤ 0.5, ns – not significant). Scale bar: (**A**–**J’**) = 10 μm.

In the current studies, we set out to determine what other functions, apart from synapse assembly, Dnrx plays in the *Drosophila* nervous system. *dnrx* mutants displayed accumulation of synaptic cargoes in motor axons that innervated the larval muscle field^3^. This phenotype is reflective of axonal transport defects. We wanted to analyze this phenotype further by looking at the segmental nerve axons that exited the ventral nerve cord (VNC) and traversed long distances before they innervated the more distal larval musculature in wild type and *dnrx* mutants (Fig. 1). We also wanted to investigate whether a defect in axonal transport might be accompanied by a disorganized axonal MT cytoskeleton^28–30^. We hypothesized that the more distal portions of axons are more sensitive to impairments of MT function and accumulation of various cargoes. We stained age-matched 3^rd^ instar wild type (Fig. 1A,A’), homozygous *dnrx* (*dnrx*−/−, Fig. 1B,B’) and *dnrx/Df(3R)5C1* (a deletion that removes *dnrx* locus; Fig. 1C,C’) larvae with anti-Synaptotagmin (Syt) (Fig. 1A–C, green) which labels synaptic vesicles^31^ and anti-Futsch (Fig. 1A’–C’, red) which labels the presynaptic MTs^26,27^. Wild type nerve fibers did not show any axonal transport defects as evidenced by anti-Syt staining (Fig. 1A), while *dnrx* mutants showed a significant accumulation of Syt puncta along the axons (Fig. 1B,C, see quantification in K, L). Moreover, axonal MTs in wild type show tightly fasciculated bundles that are arranged in parallel arrays (Fig. 1A’), while in *dnrx* mutants, axonal MT displayed a distorted and ruffling morphology and defasciculation (Fig. 1B’,C’). Next we wanted to study the effects of knocking down Dnrx in all neurons using pan-neuronal *elav-Gal4/UAS-dnrx-RNAi* (Fig. 1D,D’) or selectively in motor neurons using *OK6-Gal4/UAS-dnrx-RNAi* (Fig. 1E,E’). Both genotypes presented axonal transport defects in the mutant larvae that were significantly enhanced than the wild type (Fig. 1K,L). While the axonal transport defects resulting from Dnrx knockdown using both *elav-Gal4* (Fig. 1D) and *OK6-Gal4* (Fig. 1E) were not significantly different (Fig. 1K,L) when compared to one another, they were found to be milder than the *dnrx* mutants (for comparison, see Fig. 1K,L). We then wanted to examine whether wild type overexpression of Dnrx either pre- or postsynaptically show any of these MT organization and/or transport defects. While postsynaptic overexpression of Dnrx in *mef2-Gal4/UAS-dnrx* larvae did not display any axonal MT disorganization or transport defects (data not shown), presynaptic Dnrx overexpression using *elav-Gal4* (Fig. 1F,F’) and *OK6-Gal4* driver (Fig. 1G,G’) led to disrupted axonal MT (Fig. 1F’,G’) and blockage of Syt (Fig. 1F,F’ and G,G’) as seen in *elav-Gal4;UAS-dnrx* and *OK6-Gal4, UAS-dnrx* larvae.

Previous studies have reported that *wishful thinking (wit*, a Type II receptor of the BMP signaling pathway) mutants also display a punctate Syt staining in motor axons^13^ (Fig. 1H,H’). Based on our recent studies that showed Dnrx and Wit function together in regulation of synaptic growth at the *Drosophila* larval NMJs^8^. Given this finding, we hypothesized that Dnrx and Wit may play a similar role in coordinating axonal MT organization and transport which may or may not be dependent on synaptic growth. We first wanted to examine the presence and/or extent of axonal MT organization and transport deficits in both loss and gain of Wit functions. We found that *wit* mutants *(wit*^*A12*^*/wit*^*B11*^*)*^14^ showed structural alteration and organization defects in MTs (Fig. 1H’) in addition to accumulation of Syt, which is consistent with previous reports^13^ (Fig. 1H,H’,K,L) and similar to those observed in *dnrx* mutants (Fig. 1K,L). However, unlike Dnrx, there were no phenotypic consequences of presynaptic Wit overexpression as seen in *elav-Gal4; UAS-wit-GFP* larvae on MT organization or transport of axonal cargoes (Fig. 1I,I’, K,L). The *elav-Gal4* driver (Fig. 1J,J’) or the *OK6-Gal4* driver (data not shown) used in this study did not display any obvious axonal transport or MT organization defects along the segmental axons. In order to rule out that Syt was not the only synaptic vesicle cargo that failed to transport along segmental axons in *dnrx* and *wit* mutants, we also immunostained these mutants with other synaptic vesicle proteins, like *Drosophila* Cysteine String Protein 2 (DCSP2)^32^ and active zone protein, Bruchpilot (Brp)^33^ together with the wild type control (Fig. S1). We found blockage of both DCSP2 and Brp in *dnrx* and *wit* mutants indicating possibly a more general defect in the transport machinery. Together, these data suggest that proper axonal MT organization and transport are dependent on both Dnrx and the BMP receptor, Wit.

### Neurexin Displays Genetic Interaction with the BMP Receptor wishful thinking

**Figure 2:**
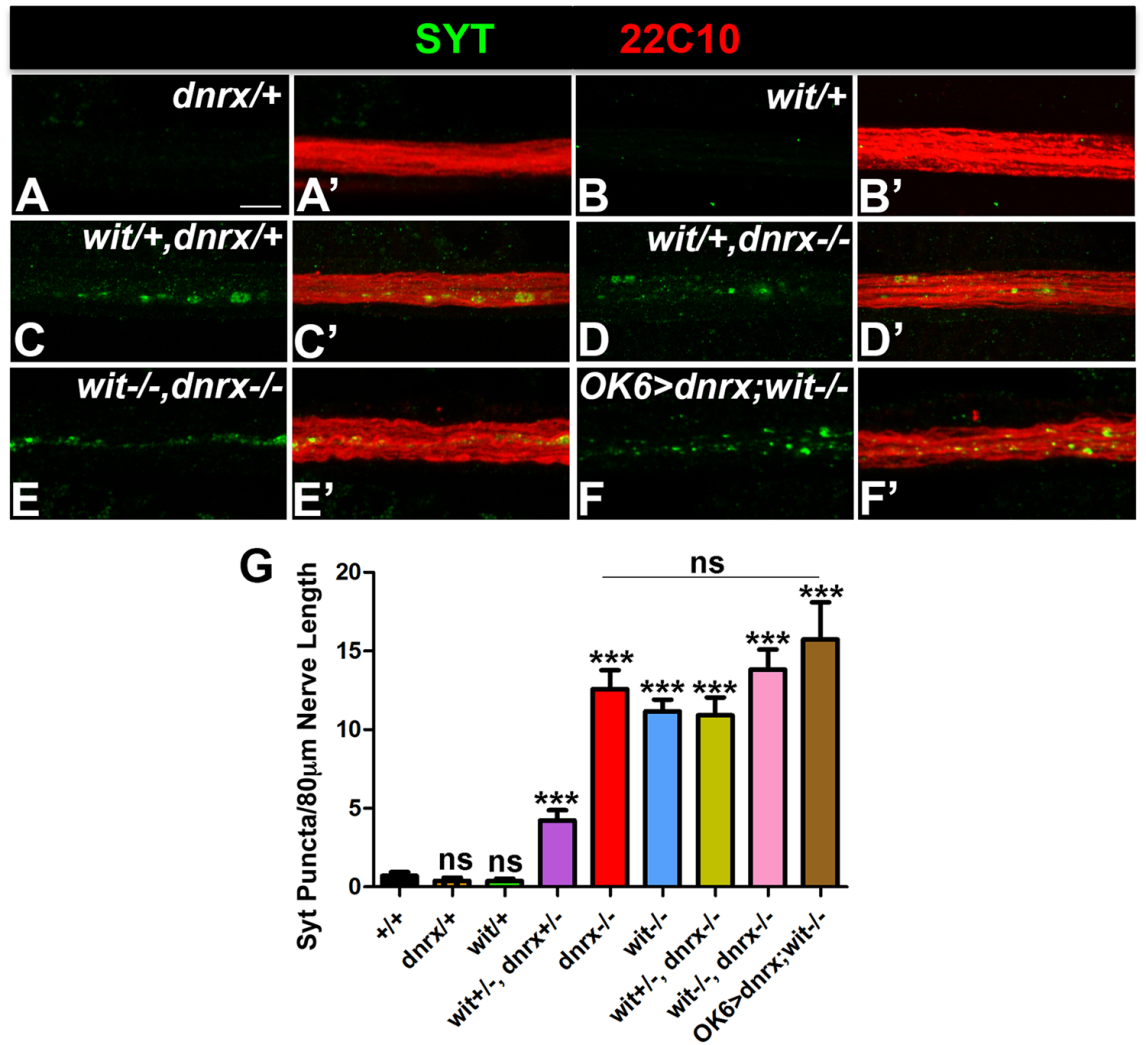
Genetic interactions between *dnrx* and *wit* and their influence on axonal transport. (**A**–**F’**) Confocal images of portions of nerve fibers showing axonal MT stained with anti-Futsch (red) and anti-Syt (green) from 3^rd^ instar larvae of indicated genotypes. (**G**) Quantification Syt puncta per 80 μm of segmental nerve 5–8 passing through A4-7 in all indicated genotypes. n = 15 animals. Error bars represent mean ± SEM (***p < 0.001, **p ≤ 0.01, *p ≤ 0.5, ns – not significant). Scale bar: (**A**–**F’**) = 10 μm.

We have shown that there are similarities in accumulation of cargoes in the axons and structural disorganization of MTs in *dnrx* and *wit* mutants. Given these observations, we wanted to further explore whether *dnrx* and *wit* display genetic interactions and likely play a role together in regulating MT organization and transport. For these genetic interaction studies, we generated various combinations of *wit* and *dnrx* mutants, including double mutants. We first tested *dnrx*/+ (Fig. 2A,A’,G) and *wit*/+ (Fig. 2B,B’,G) heterozygotes and found them to be similar to wild type (Fig. 2G). Interestingly, transheterozygous combination of *witx*/+, *dnrxx*/+ (Fig. 2C,C’,G) showed a significant accumulation of Syt puncta which is not observed in wild type controls (Fig. 2G) or individual heterozygotes (Fig. 2A,B,G). Loss of one copy of *wit* in *dnrx* (Fig. 2D,D’,G) mutants did not show an increase in Syt puncta along the motor axons compared to *dnrx* or *wit* mutants (Fig. 2G). Double mutants of *wit,dnrx* (Fig. 2E,E’,G) displayed no significant difference in terms of accumulation of Syt puncta along axons compared to *wit* mutants (Fig. 2G). Interestingly, other measurement of Syt puncta, like area and intensity was found to be similar in *wit* and *dnrx* single and double mutants (Fig. S2). These genetic interaction data confirm that Dnrx functions together with Wit for regulating axonal transport. We also studied the effect of *wit* loss with Dnrx overexpression using motor neuronal driver, *OK6-Gal4* (Fig. 2F,F’). We found it to be comparable to the double loss of *wit,dnrx* double mutants (Fig. 2G). Finally, various genetic combinations of *wit,dnrx* (Fig. 2D’,E’) mutants also show MT structural alteration and organization defects as observed in *dnrx* and *wit* mutants (Fig. 1). These data suggest that *wit* and *dnrx* display genetic interactions and coordinate proper axonal MT organization and transport during nervous system development.

### Ultrastructural Defects in Axonal Microtubules in *dnrx* and *wit* Mutants

**Figure 3:**
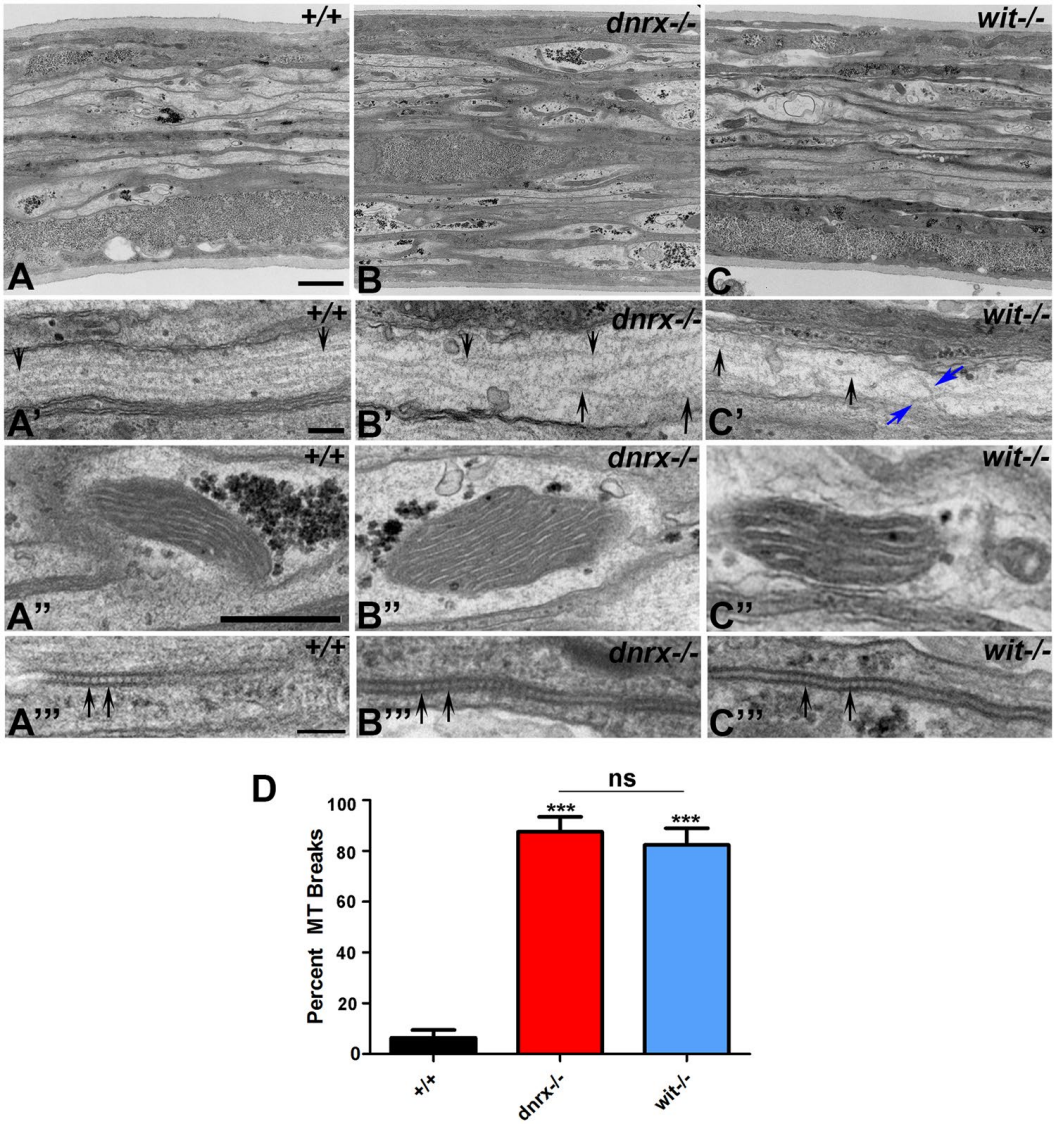
Ultrastructural changes in axonal microtubule organization in *dnrx* and *wit* mutants. (**A**–**C’**) Longitudinal sections at low (**A**–**C**) and high magnifications (**A’**–**C’**) of segmental nerves of +/+ (**A**,**A’**), *dnrx* (**B**,**B’**) and *wit* (**C**,**C’**) mutants. Black arrows (**A’**–**C’**) represent axonal MT filaments in indicated genotypes and blue arrows (*wit* mutants, **C**) point to intersecting MT filaments. (**A”**–**C’”**) Higher magnification images showing mitochondria (**A”**–**C”**) and septate junctions (**A’”**–**C’”**) in wild type (**A”**,**A’”**), *dnrx* (**B”**,**B’”**) and *wit* (**C”**,**C’”**) mutant nerves. (**D**) Quantification of percent MT breaks in axons of wild type, *dnrx* and *wit* mutants. Scale bars: (**A**–**C**) = 1 μM, (**A’**–**C’**) = 200 nm, (**A”**–**C”**) = 600 nm and (**A’”**–**C’”**) = 200 nm. n = 5 animals/genotype. Error bars represent mean ± SEM (***p < 0.001, **p ≤ 0.01, *p ≤ 0.5, ns – not significant).

*dnrx* and *wit* mutants have distorted axonal MT organization and ruffling morphology that is visualized by anti-Futsch immunostaining. The fluorescence intensity ratio of Futsch/Hrp in *wit* and *dnrx* mutants did not show any significant difference in segmental axons when compared to wild type (Fig. S2). We were limited by not having a clear quantifiable parameter to analyze MT disorganization by light microscopy. Given this limitation, we wanted to study the structural organization of MTs at a high resolution in these mutants compared to the wild type control. To achieve this, we analyzed longitudinal sections of the peripheral nerve fibers of wild type (Fig. 3AA’”), *dnrx* (Fig. 3B-B”’) and *wit* (Fig. 3C-C”’) mutant larvae using transmission electron microscopy (TEM) to study the arrangement and organization of MTs. Longitudinal sections of nerve fibers at lower magnification (Fig. 3A–C) of all three genotypes showed similar overall organization with axons separated by glial processes (represented by the darker cytoplasmic areas). *dnrx* and *wit* mutants occasionally displayed thinner outermost membrane layer (Fig. 1B,C), the neural lamella^34,35^, that provides mechanical support to the axon and was not significantly different than wild type (data not shown). However, at higher magnification, there was a noticeable difference in axonal MT organization in *dnrx* (Fig. 3B’) and *wit* (Fig. 3C’) compared to wild type controls (Fig. 3A’). Wild type axons have long parallel arrays of MT filaments (arrows, Fig. 3A’) with consistent spacing between them. This is similar to what is observed by anti-Futsch immunostaining (Fig. 1A’). *dnrx* (Fig. 3B’) and *wit* (Fig. 3C’) mutants displayed shorter, broken MT filaments (black arrows, Fig. 3B’,C’) that are disarrayed and sometimes even appear to crisscross as filaments intersect each other (blue arrows, Fig. 3C’). These mutant phenotypes are consistent with those seen by light microscopy (Figs 1 and 2). However, given the fragility of MTs under the TEM processing conditions, we wanted to rule out that the MT fragmentation observed in *dnrx* and *wit* mutants (Fig. 3B’,C’, respectively) is not due to preservation/fixation artifact. We therefore looked at the ultrastructural morphology of other organelles, e.g., mitochondria (Fig. 3A”–C”) and SJs^34–36^ (Fig. 3A’”–C’”) across all three genotypes. The morphology of these structures (A”–C” and A’”–C’”) was preserved and consistent across the genotypes. Quantification of MT breaks from longitudinal sections of *dnrx* and *wit* mutants (Fig. 1D) revealed significantly higher percentage of breaks compared to the wild type axons. These data demonstrate that loss of Dnrx and Wit are required for proper organization of MTs along the segmental axons.

### *dnrx* and *wit* mutant larvae Show Larval Locomotor Deficits

**Figure 4:**
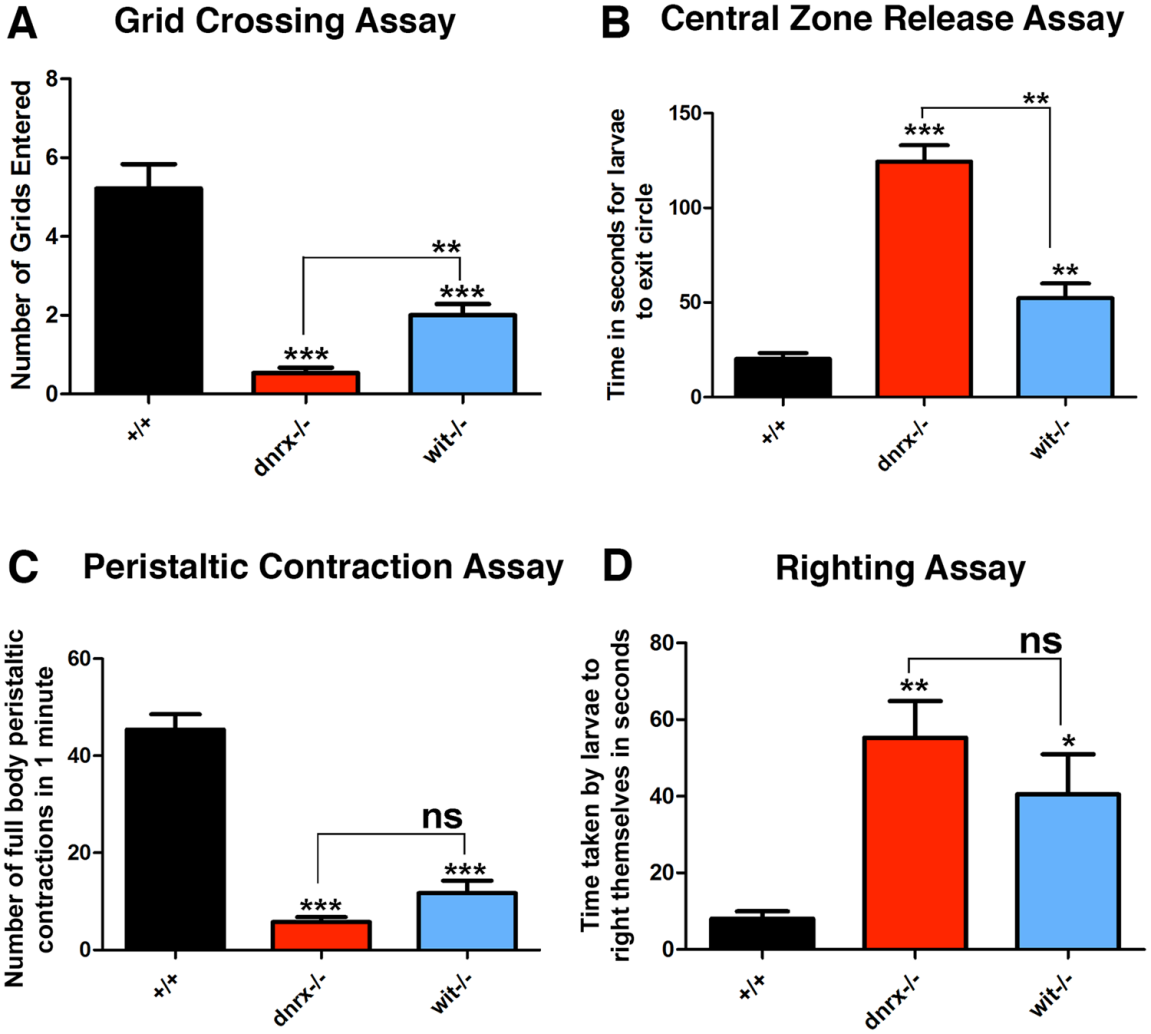
*dnrx* and *wit* mutants show defects in larval locomotion. (**A**–**D**) Wild type, *dnrx* and *wit* mutant larval locomotor behavior assayed by measuring the number of 0.5 cm^2^ grids crossed in 30 seconds (**A**), time taken in seconds for larvae to exit a circle of 1.5 cm in diameter (**B**), number of full body peristaltic contractions in 1 minute (**C**) and time taken in seconds for larvae to right themselves when turned on their dorsal surface (**D**). n = 50 animals/genotype. Error bars represent mean ± SEM (***p < 0.001, **p ≤ 0.01, *p ≤ 0.5, ns – not significant).

Mutations in genes with defects in MT organization and/or axon transport often display a range of behavioral deficits in the locomotor activities of larvae^28^. Given the impaired axonal transport and disorganized MT cytoskeleton in the segmental nerves of *dnrx* and *wit* mutants (Figs 1–3), we wanted to examine the locomotor behavior in these mutant larvae compared to their wild type counterparts. We performed behavior assays to examine various locomotion activity, patterns and rhythms. We used a grid crossing assay (Fig. 4A) where larvae are placed on a horizontal agar surface and the number of 0.5 cm^2^ grids crossed are counted within a timeframe of 30 seconds^3^. We found that both *dnrx* and *wit* mutants showed significantly fewer number of grids entered compared to wild type larvae (Fig. 4A). In the next assay, larvae were placed at the center of a circle termed as the “release zone”^37^ (Fig. 4B). Typically, wild type larvae display a dispersal behavior where they quickly move away from their point of origin while *dnrx* and *wit* mutants took significantly longer time to exit the circle (Fig. 4B). In both instances (Fig. 4A,B), *dnrx* mutant larvae were found to be significantly slower than *wit* mutants.

Another major larval behavior is forward locomotion, which is achieved by sequential contraction of segments driven by waves of peristalsis that travel from tail (posterior) to head (anterior). We next examined the rhythmic peristaltic contraction behavior in *dnrx* and *wit* mutants compared to wild type larvae (Fig. 4C). We found that mutants of *dnrx* and *wit* had significantly fewer peristaltic contractions compared to wild type larvae (Fig. 4C). The last locomotion analysis performed was the “righting assay”^38^. In this assay, wild type, *dnrx* and *wit* mutant larvae were placed on their dorsal side and the time taken to return to their ventral (crawling position) side was measured (Fig. 4D). Both *dnrx* and *wit* mutants took significantly longer time to return to their crawling position compared to wild type larvae (Fig. 4D). Taken together, these datasets indicate that both *wit* and *dnrx* mutant larvae display severely impaired locomotor behavior consistent with axonal MT disorganization and axonal transport, as has been observed in other MT-related mutants.

### Axonal Transport Defects in *futsch* Mutants and Genetic Interactions with *dnrx*

**Figure 5:**
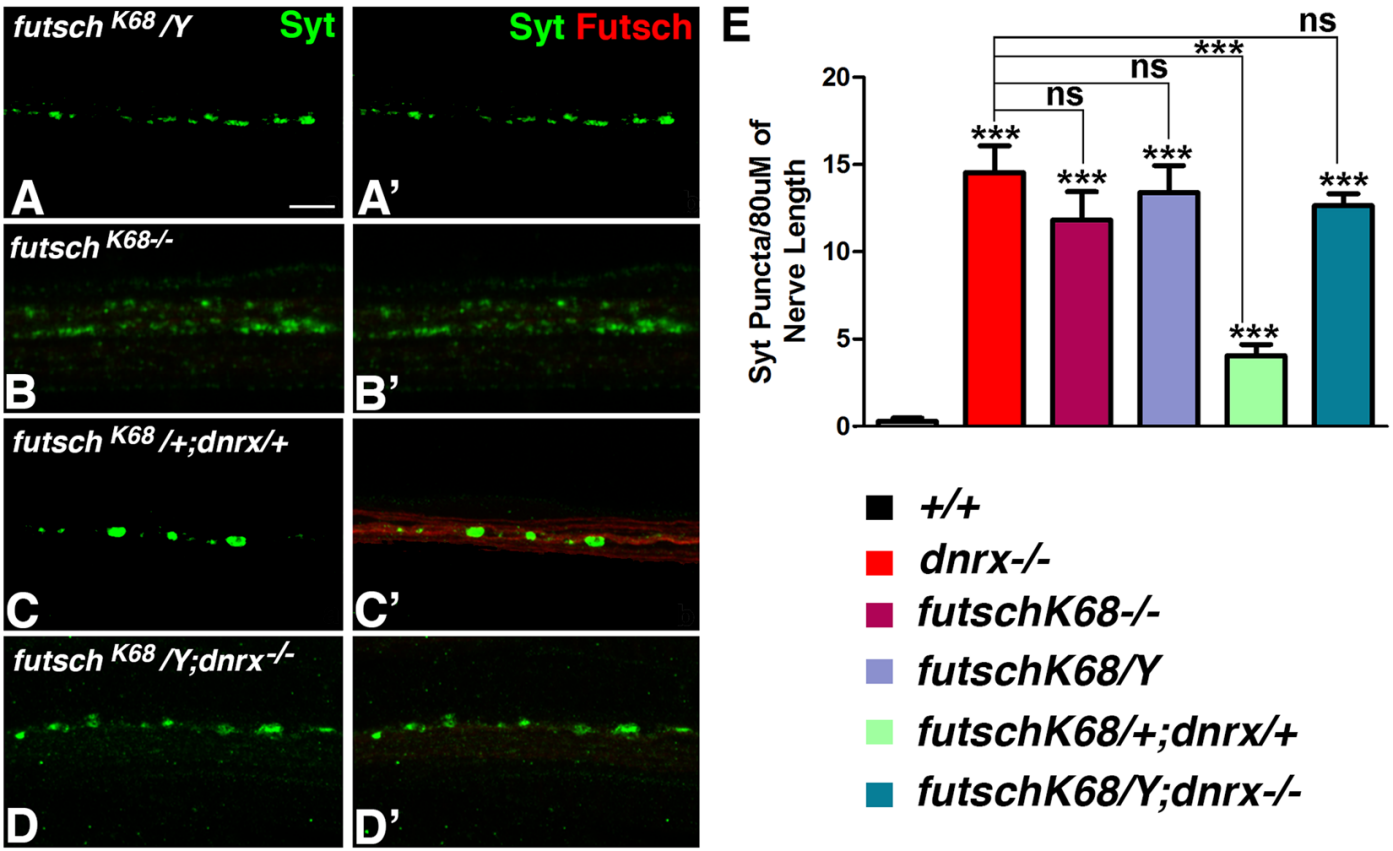
Axon Transport Defects in *futsch* Mutants and Genetic Interactions between *dnrx* and *futsch*. (**A**–**D**’) Portions of segmental nerves labeled with anti-Syt (green) and anti-Futsch (red) in indicated genotypes. (**E**) Quantification of Syt puncta along 80 μM of segmental nerves of indicated genotypes. Error bars represent mean ± SEM (***p < 0.001, **p ≤ 0.01, *p ≤ 0.5, ns – not significant). Scale bar: (**A**–**D**’) = 10 μm.

Previous studies have implicated *Drosophila* microtubule associated protein, Futsch, in the BMP pathway in regulating MT organization and stability^18,20^. Similar to Wit, both Dnrx and Futsch are found in both the presynaptic area and along axons. Since the presynaptic MT organization as visualized by anti-Futsch immunostainings (Fig. 1) and by high resolution TEM (Fig. 3) was affected in *dnrx* mutants, we wanted to further study the relationship between Dnrx and Futsch. More specifically, we wanted to determine: 1) whether there are axonal transport defects in *futsch* mutants similar to *dnrx* mutants, and 2) whether *futsch* and *dnrx* display genetic interactions. *futsch*^*K68*^*/Y* (Fig. 5A,A’) and *futsch*^*K68*^*/futsch*^*K68*^ (Fig. 5B,B’) motor neurons show loss of Futsch expression in axonal MT (Fig. 5A’,B’, respectively) and pronounced vesicular Syt aggregates along the axons (Fig. 5A,B, respectively). This is similar to *dnrx* mutants (Figs 1B,C, 5E). In order to test for genetic interactions between *dnrx* and *futsch*, we generated transheterozygous combinations of *futsch*^K68^/+; *dnrx*/+ and double mutants of *futsch*^*K68*^*/Y;dnrx*. Transheterozygous *futsch*^K68^/+; *dnrx*/+ larvae showed a significant increase in axonal Syt puncta (Fig. 5C,E) compared to control wild type (Fig. 5E) or individual heterozygotes (*data not shown*) suggesting that loss of *futsch* and *dnrx* may affect a common pathway. Double mutants of *futsch*^K68^*/Y; dnrx* (Fig. 5D,E) did not show any additive increase in the vesicular Syt accumulation than individual single mutants. *futsch* mutants also display significantly increased Brp-positive puncta along distal segmental axons (Fig. S3) compared to their wild type counterparts. These data show that *dnrx* and *futsch* display genetic interactions, and also regulate MT organization and axonal transport during nervous system development.

### Consequences of Postsynaptic Upregulation of BMP ligand, Glass Bottom Boat, in *dnrx* Mutant

**Figure 6:**
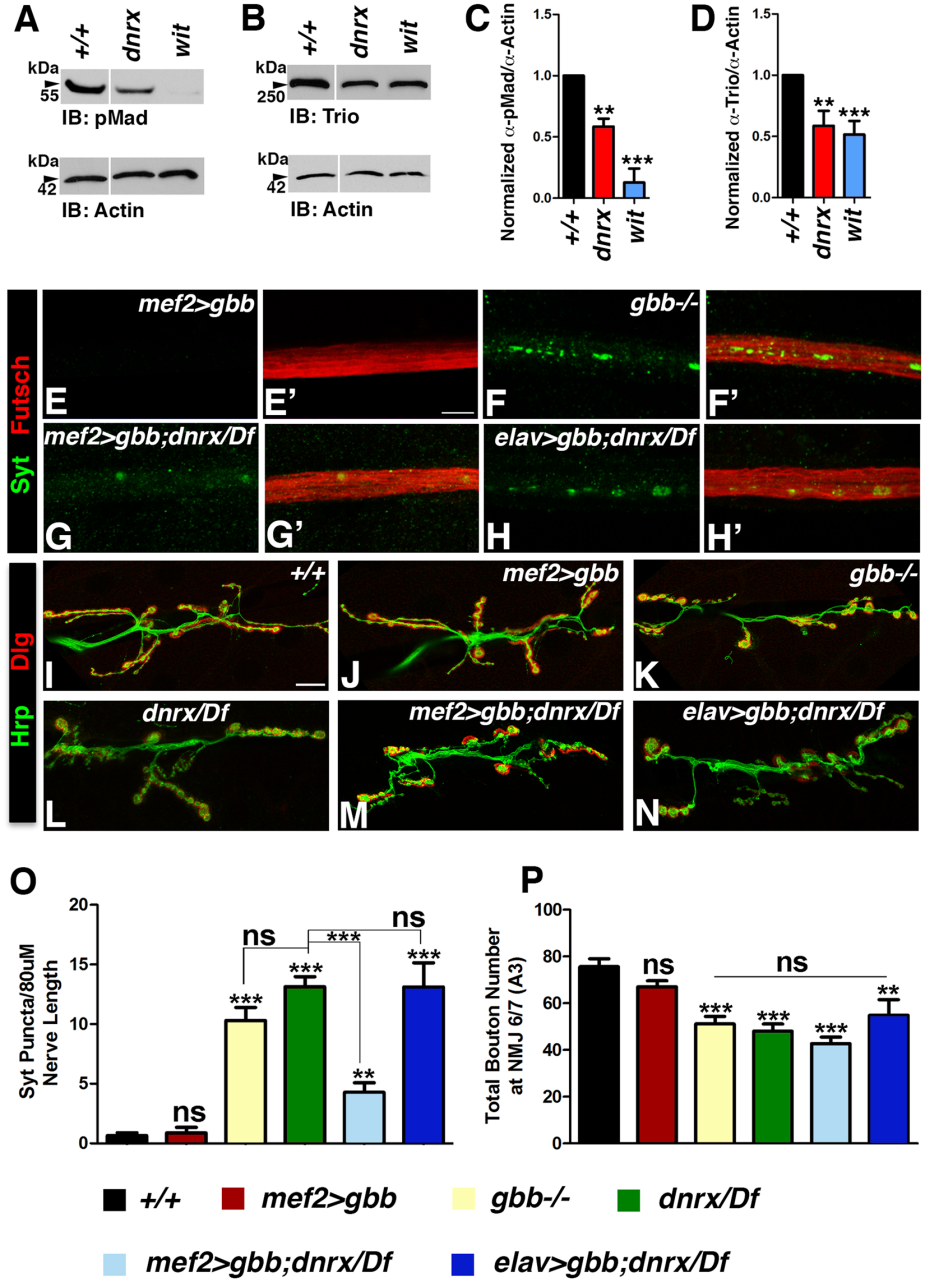
Postsynaptic Overexpression of Gbb in *dnrx* mutants partially rescues the axonal transport defects. (**A**–**D**) Immunoblots of brain lobes/VNC lysates from wild type*, dnrx* and *wit* mutants showing levels of phosphorylated Mad (pMad, 55 kDa, **A**) and the cytoskeletal RhoGEF, Trio (250 kDa, **B**), together with their corresponding Actin levels as loading controls. (Note the breaks in panels separated by white space are due to removal of irrelevant lanes, see Fig. S4). Ratio of band intensity was quantified by measuring levels of pMad to Actin (**C**) and Trio to Actin (**D**) in indicated genotypes. (**E**–**H’**) Confocal images of portions of segmental nerves from 3^rd^ instar larvae stained against Futsch (red) and Syt (green) in various genotypes. (**I**–**N**) Confocal images of larval NMJ synaptic boutons stained with antibodies against Dlg (red) and Hrp (green) in indicated genotypes. (**O**,**P**) Quantification of Syt puncta (**O**) and total bouton numbers in NMJ 6/7 of A3 (**P**) in indicated genotypes. n = 15 animals/genotype in (**E**–**N**). Error bars represent mean ± SEM (***p < 0.001, **p ≤ 0.01, *p ≤ 0.5, ns – not significant). Scale bars: (**A**–**H’** and **I**–**N**) = 10 μm.

Having established that Dnrx shows phenotypic similarities and genetic interactions with the type II BMP receptor, Wit, we next wanted to investigate if the loss of Dnrx had any impact on the immediate downstream effectors of the Wit pathway. For these studies, we examined whether the classical readout of the BMP pathway, namely pMad levels are affected in *dnrx* mutant VNC. We carried out pMad immunoblot analysis of brain lobe/VNC lysates (Fig. 6A) from wild type*, dnrx* and *wit* mutants. As expected, *wit* mutants showed a severe reduction in pMad levels (Fig. 6A,C). We observed a significant reduction of pMad in *dnrx* (Fig. 6A,C) mutants compared to wild type (Fig. 6A,C). We next tested for another downstream component of the BMP cascade, the Rho type guanyl nucleotide exchange factor (GEF) Trio^39^, by immunoblotting of brain lobes/VNC extracts from wild type*, dnrx* and *wit* mutants. The levels of Trio were significantly reduced in both *dnrx* and *wit* mutants (Fig. 6B,D). Immunoblot analysis with anti-Actin served as loading controls (Fig. 6A,B). These data reveal that loss of Dnrx leads to reduced levels of the pMad and Trio.

To further gain mechanistic insights into the axonal transport deficits of *dnrx* mutants and establish the involvement of the BMP signaling pathway, we next designed experiments to bypass the requirement of Dnrx in *dnrx* mutants. We did this by postsynaptic overexpression of the Wit ligand, Gbb. We also used these methods to determine to what extent, if any, the axonal transport phenotypes in these mutants could be rescued. For these studies, we utilized the *UAS-gbb* overexpression using *mef2-Gal4* first. We wanted to confirm if *mef2-Gal4;UAS-gbb* overexpression and *gbb*^*1*^*/gbb*^*4*^ mutants show any axonal transport defects as revealed by accumulation of Syt puncta along segmental axons. While overexpression of Gbb in *mef2-Gal4;UAS-gbb* did not cause any Syt accumulation along axons (Fig. 6E,E’,O), *gbb*^*1*^*/gbb*^*4*^ mutants displayed significant accumulation of Syt puncta along axons^18^ (Fig. 6F,F’,O). Next, we tested if overexpression of *UAS-gbb* postsynaptically could rescue *dnrx* mutant phenotypes. As *dnrx* homozygous mutants in *mef2-Gal4;UAS-gbb* background caused lethality, we used *dnrx/Df(3R)5C1* for generating *dnrx* null background^3^. *dnrx/Df(3R)5C1* showed similar axonal transport phenotypes as *dnrx* mutants (Fig. 1C,K,L). *mef2-Gal4/UAS-gbb* in the mutant background of *dnrx/Df(3R)5C1* (Fig. 6G,G’) showed a significant improvement in axonal transport defects compared to *dnrx/Df(3R)5C1* alone (Fig. 6O) [*** for difference between *dnrx/Df* and *mef2-Gal4/UAS-gbb;dnrx/Df(3R)5C1* rescue bar heights]. However, the rescue was not complete [** for difference between the rescue genotype and the wild type bar heights]. We also examined the pre-synaptic overexpression of Gbb using *elav-Gal4* in the mutant background of *dnrx/Df(3R)5C1* to see if that affects the phenotype. The axonal transport phenotype was not rescued in *elav-Gal4;UAS-gbb;dnrx/Df(3R)5C1* mutant larvae (Fig. 6H,H’,O). This data reveals that Dnrx and the Gbb-dependent BMP signaling pathway display phenotypic similarities with respect to axonal MT organization and transport and that overdriving the BMP pathway partially rescues the axonal transport defects in *dnrx* mutants.

Since Dnrx and the BMP signaling pathway are implicated in regulating synaptic growth, we wanted to examine if transport and synaptic growth are inter-related. Specifically, we asked whether the pre- or postsynaptic overexpression of Gbb will rescue the bouton growth phenotype of *dnrx* mutants? In order to analyze synaptic growth, larval NMJs were immunostained with antibodies against postsynaptic density protein, Discs large (Dlg)^40^, and the neuronal marker Hrp. As presented here (Fig. 6I–N, P) and reported earlier, *dnrx* (Fig. 6L,P) and *gbb* (Fig. 6K,P) mutants show synaptic undergrowth at the larval NMJ^3,8,11,15^ compared to their respective wild type control. Postsynaptic overexpression of Gbb in *mef2-Gal4/UAS-gbb* (Fig. 6J,P) did not show any significant differences in bouton numbers compared to wild type. Postsynaptic overexpression of Gbb in *mef2-Gal4/UAS-gbb;dnrx/Df(3R)5C1* (Fig. 6M,P) as well as presynaptic overexpression of Gbb in *elav-Gal4;UAS-gbb;dnrx/Df(3R)5C1* (Fig. 6N,O) mutants failed to rescue the synaptic undergrowth phenotype seen in *dnrx/Df(3R)5C1* mutants (Fig. 6L,P). These datasets suggest that regulation of axon transport deficits in *dnrx* mutants might be distinct from regulation of synaptic growth during larval NMJ development, and that Dnrx plays a much broader role in axonal transport and synaptic development.

## Discussion

Neurexins are some of the most extensively studied synaptic cell adhesion molecules for their role in synapse formation and function^3,4,8,10–12^. In *Drosophila, dnrx* mutants have reduced synaptic growth. This reduced synaptic growth is similar to what we observe in the BMP signaling mutants: *wit, gbb, mad* and *trio*^3,8,10,13–15,39,41^. Our recent studies have shown that *dnrx* regulates components of the BMP signaling pathway and directs synaptic growth and cytoarchitecture in conjunction with *dnlg1* and *wit*^8^. The data presented here show that Dnrx engages in a complex molecular machinery with the BMP receptor Wit, the ligand Gbb and other downstream effectors to allow smooth axonal transport and proper MT organization.

Both *dnrx* and *wit* mutants display phenotypic similarities with axonal MT organization and transport of various cargo along the segmental nerves (Figs 1; S1; Table S1). It is interesting to note that axonal MT and transport phenotypes resulting from gain of Dnrx function are similar to loss of function mutants. This suggests that a fine balance in levels of Dnrx is important, and either too little or too much of it is detrimental for MT organization and the transport machinery. Defects in axonal transport resulting from cell type specific reduction in motor neurons using *dnrx-RNAi* confirmed that the axon transport defect is autonomous to motor neurons (Fig. 1). The defects resulting from the Dnrx knockdown, however, were milder than *dnrx* loss-of-function possibly due to penetrance or expressivity factors. The Gal4-only controls (*elav-Gal4* and *OK6-Gal4*) did not display any axonal transport or MT organization defects further confirming the specificity of the *dnrx* phenotypes. It is also worth noting that gain of Wit did not disrupt MT organization or axonal transport.

The genetic interaction between *dnrx* and *wit* suggest that these proteins regulate axonal MT organization and transport (Fig. 2). Our recent studies have also showed that Dnrx and Wit coordinate synaptic organization and growth^8^, which suggests that these proteins are needed for multiple processes during nervous system development. The ultrastructural phenotypes in *dnrx* and *wit* mutants (Fig. 3) reveal a structural disorganization in the arrangement of MT filaments along the axons. These datasets showed that there is indeed disorganization in MT morphology in our mutants and these findings are not artifacts from the altered localization of Futsch (Figs 1 and 2).

Both *dnrx* and *wit* are presynaptic proteins and their loss shows a multitude of defects at the axonal, cytoskeletal and synaptic levels. It was therefore important to examine the range of behavioral deficits in these mutant larvae. *dnrx* and *wit* mutants displayed defects both in peristalsis and locomotor behaviors. These behaviors are attributable, most likely at least in part, to the defects in axon transport, MT organization and possible dysfunction of the NMJ synapses. Behavior in animals could be a composite of defects at many levels of integration at the CNS, PNS, NMJ and circuits responsible for producing rhythmic patterns of movement and general locomotion^42^. Defects in both peristalsis and wandering behavior in *cysteine string protein (csp)* mutant larvae, for example, are thought to be due to motor defects at the NMJ and at the levels of CNS output generation^43^. The locomotor behaviors (Fig. 4A,B) observed in *dnrx* and *wit* mutants also indicate that these proteins might play a boarder role in organization of motor circuits.

Recent studies have implicated BMP signaling in the regulation of synaptic strength, axonal MT organization and transport^17,18,20^. While our studies point to the reliance of MT organization and axonal transport on BMP signaling, there are other studies that show disruption of axonal transport perturbing BMP signaling contributes to synaptic abnormalities in neurodegenerative diseases^44^. It is important to note that mutations that fall under the categories of both positive regulators of BMP, like *dnrx*^8^ and negative regulators of BMP such as, Spicthyin and Spartin^18,20^ both lead to MT disorganization. It is, therefore, interesting that while Spichthyin and Spartin function as negative regulators of the BMP signaling by regulating BMP receptor traffic and modulate regulation of MT cytoskeleton, loss of these proteins does not show impaired axonal transport^18,20^. Dnrx loss results in disorganized MTs, but unlike Spichthyin and Spartin, loss of Dnrx shows transport defects. These findings suggest that MT cytoskeletal organization could be compromised by both positive and negative regulation of BMP signaling.

The regulation of microtubule stability is essential for axonal transport. The microtubule-associated protein, Futsch, recently implicated in the BMP pathway^18,20^, has a role in the stabilization of MTs^26,27^. *futsch* mutants display axonal transport defects consistent with altered MT organization similar to *dnrx* and *wit* mutants (Fig. 5). A previous report, however, showed that *futsch* mutants do not show defects in axonal transport of Brp^45^. We examined *futsch* mutants for accumulation of Brp puncta along axons using our experimental parameters. We found mild, yet significant, increase in accumulation of Brp along the axons in *futsch* mutants. Our studies are not completely in agreement with the previous report^45^. The discrepancies between the studies could be possibly due to: (1) differences in quantification parameters (Brp area/Hrp area^45^ as opposed to number of Brp puncta along nerve length); (2) difference in segmental nerves analyzed (our analysis was restricted to segmental nerves 5–8); and (3) the region of nerve fiber analyzed (proximal or distal). It is also worth noting that our light microscopy studies showed Syt and DCSP2 aggregation along mutant axons did not always co-localize with Brp puncta (Fig. S1). These observations are consistent with recently published report that showed Syt co-traffic less efficiently with active zone scaffold proteins Brp and Basoon^46^. Collectively, these findings suggest the possibility of the presence of distinct transport mechanisms for different synaptic cargoes. Future studies will be aimed at addressing the dynamics of axonal membrane traffic in mutants of *dnrx* and BMP pathway, and whether some synaptic proteins are co-transported and others are not, or whether transport mechanisms are able to discriminate synaptic cargo as it is transported along the axons.

*futsch* and *dnrx* interact genetically as double heterozygotes display axonal transport defects consistent with dose-dependent genetic interactions (Fig. 5). This data further corroborates the idea that Dnrx is not only necessary for trans-synaptic adhesion and apposition of the pre- and post-synaptic compartments, but also for maintaining the stability of the BMP receptor Wit for optimal BMP signaling^8^. Future studies will be aimed at addressing the link between Dnrx and Futsch. Dnrx is a transmembrane protein with a PDZ domain binding motif^3^ and Futsch is a MT-binding protein^26^. Both of these proteins are highly expressed along the axons and could potentially exist as a complex. It is also possible that Dnrx might interact with MT motor proteins such as Kinesin or Dynein. The Kinesin superfamily protein, KIF 17, was previously shown to interact with PDZ domain of mLin-10 (Mint1/X11) to transport NMDA receptor 2B. mLin-10 is part of a larger protein complex that includes mLin-2 (Cask) and mLin-7 (Veli)^30^. It is likely that the intracellular PDZ domain of Dnrx is capable of recruiting protein complexes such as these to form a link between axonal membranes to the axonal MT cytoskeleton.

While it was not a surprise that levels of the downstream effectors of the BMP cascade, pMad and Trio, would be reduced in *dnrx* mutant BL/VNC (Fig. 6) given that our previous studies showed reduced levels of Wit, Tkv and pMad at the *dnrx* mutant NMJ synapses^8^. It is interesting that postsynaptic overexpression of the BMP ligand, Gbb, partially rescues the axonal transport phenotypes in *dnrx* mutant background but not the NMJ bouton undergrowth phenotype (Fig. 6). This indicates that distinct regulatory mechanisms might be involved in proper axonal MT organization and transport and growth during NMJ development, which are still not well understood. While this finding may suggest that Wit/Gbb signaling does not require Dnrx to regulate axon transport, it is important to note that the rescue of the axonal transport phenotype was partial and did not reach wild type levels, suggesting that Dnrx is required to allow optimal levels of axonal transport to occur. This finding also indicates: (1) that possibly it is the retrograde transport that gets somewhat corrected while there might still be defects in anterograde transport of cargoes in *dnrx* mutants; (2) that BMP signaling solely is not responsible for trafficking of cargoes and overall MT health in *dnrx* mutants; and (3) that there might be additional proteins involved in forming a cascade to link Dnrx from axonal membrane to MT cytoskeleton. Given our findings as reported here on *Drosophila* Nrx, it would be of immense interest to investigate whether mammalian Neurexins will have similar functions separate from their role in synapse formation and their functional modulation, and also axonal microtubular organization. Future studies will further address other aspects of Neurexin functions in the nervous system.

## Methods

### Fly Stocks

The fly strains used in this study were isogenized *w*^*1118*^ *Canton-S* line (a gift from V. Budnik) used as the *WT* control, *dnrx*^*273*^, *UAS-dnrx*^3^, *UAS-gbb, gbb*^*1*^ and *gbb*^*4*^,^47^ and *UAS-wit-GFP* (a gift from M. O’Connor). All other fly stocks were obtained from Bloomington Stock Center, Indiana. *UAS-dnrx-RNAi* was obtained from Vienna Drosophila Resource Center. All flies were maintained at 22 °C, 50% humidity and with a 12-hour light/dark cycle. To avoid over-crowding, all fly lines for various phenotypic analyses were set up using 10 females and 5 males and transferred every 24 hours into fresh media.

### Immunohistochemistry and Confocal Imaging

Wandering third-instar larvae for various genotypes were dissected and fixed in Bouin’s fix for 15 minutes and processed simultaneously as previously described^11^. Confocal images of all genotypes of larvae belonging to the same experimental group were acquired using same settings with a Zeiss LSM710 confocal microscope.

The following primary antibodies were used: FITC-conjugated anti-HRP (1:250, Jackson ImmunoResearch laboratories), Rabbit anti-Syt^31^ (1:1000, H. Bellen, Baylor College of Medicine), mouse monoclonal anti-Dlg (1:1500, 4F3), anti-Brp (1:250, NC82), anti-DCSP2 (1:250, 6D6) and anti-Futsch (1:1000, 22C10) obtained from Developmental Studies Hybridoma Bank (DSHB), University of Iowa. Anti-Hrp conjugated to Alexa 488 or 568 were used at 1:250. Secondary antibodies conjugated to Alexa 488, 568, and 647 (Invitrogen-Molecular Probes) were used at 1:200.

### Electron Microscopy

Ultrastructural analyses of third-instar larval peripheral nerves were processed for TEM as previously described^8^. 5 larvae were processed for EM analysis from each of the genotypes shown in Fig. 3 and at least 50 longitudinal sections of segmental nerves from each of the specified genotypes were analyzed to show a representative phenotype and for quantification of MT breaks.

### Immunoblotting Analysis

3^rd^ instar larval VNC and brain lobes without any attached imaginal discs were homogenized in ice-cold RIPA buffer^8^. The supernatants with equal amounts of proteins from each genotype were separated on SDS-PAGE for immunoblotting with respective antibodies. Each experiment was done independently three times and the most representative blots are shown.

Primary antibodies used for immunoblotting were: anti-Trio (1:250; 9.4A, DSHB), anti-Smad3 (1:150, Abcam), and anti-βActin (1:10,000, 4967S, Cell Signaling).

Composition of RIPA buffer: 150 mM sodium chloride, 1% Igepal CA630, 0.5% Deoxycholate, 0.1% SDS and 50 mM TrisHCl, pH 8.0.

### Larval Locomotion Assays

The larval locomotion assay was performed as described previously^3,37,38,48^ with minor modifications. Number of larvae (n) analyzed from the specified genotypes were >50. Larvae were first washed briefly in distilled water to remove any traces of food before performing various assays. Each larva was acclimatized to the test plate for 1 minute prior to testing. 5 trials per larvae were conducted. Statistics was performed using Graphpad PRISM software as described in the following section.

#### Grid crossing assay

Individual larvae were placed in the center of a 145-mm diameter Petri Dish, with 3% non-nutritious agar previously poured and allowed to harden covering a graph paper at the bottom with 0.5 cm^2^ grids marked. The number of grid line crossings within a 30 second time window was recorded five times per larva.

#### Central zone release assay

A circular white card 1.5 cm in diameter was taped to the bottom of the dish to mark the central release zone. 5–10 animals were placed at the center of the release zone. The time taken for each animal to exit the release zone was recorded.

#### Peristalsis contraction assay

Full body peristalsis contractions (full posterior to anterior movement = 1 contraction) were counted for each larva in one minute while observing under a dissection microscope.

#### Righting assay

Larvae were placed on the agar plate for one minute to allow them to equilibrate to the new environment. They were then turned on their dorsal surfaces with a fine brush and the time taken to return to their ventral crawling position was noted.

### Quantification and Statistical Analysis

Bouton number quantifications were performed at muscles 6/7 of abdominal segment 3 (A3) by staining of the body wall muscle preparations with anti-HRP and anti-Dlg. Axonal Syt accumulations were quantified in regions of segmental nerve axons as they passed through A4-A7. The number of Syt and Brp puncta along these axons were counted per 80 μm of nerve length or nerve area (μm^2^).

ImageJ was used for quantification of band intensities of western blots from three independent experiments. The intensity of the bands of interest were divided by their respective Actin protein to control for any possible unequal loading. ImageJ was also used for quantification of fluorescence intensity measurements as previously reported^8^.

All statistical analyses were performed using the Graphpad PRISM software and data are presented as mean ± SEM. Statistical significance was determined by one-way ANOVA followed by post hoc Tukey’s multiple comparison test and Student’s t-test. Error bars represent mean ± SEM (***p < 0.001, **p ≤ 0.01, *p ≤ 0.5, ns – not significant).

## Electronic supplementary material

Supplemental Information

## Data Availability

The authors declare that all data generated or analyzed in this study are available within the article. The data that support the findings of this study are available from the corresponding author upon reasonable request.
