# Supplementary material for: Coordinated Regulation of Axonal Microtubule Organization and Transport by Drosophila Neurexin and BMP Pathway
Source: Sci Rep. 2018 Nov 26;8:17337. doi: 10.1038/s41598-018-35618-7 (PMC6255869; doi:10.1038/s41598-018-35618-7)
Supplement: Supplementary file 1 — Supplemental Information [file 41598_2018_35618_MOESM1_ESM.pdf]

SUPPLEMENTAL INFORMATION

Coordinated Regulation of Axonal Microtubule Organization and Transport by *Drosophila* Neurexin and BMP Pathway

Swati Banerjee[1]* and Maeveen Riordan[1,2],

[1]Department of Cellular and Integrative Physiology,
Long School of Medicine, University of Texas Health,
7703 Floyd Curl Drive, San Antonio, TX 78229, USA

[2]Present Address: University of Colorado School of Medicine, 12631 E. 17th Avenue B177, Aurora, CO 80045, USA

Running Title: Neurexin in Microtubule Organization and Transport

Key words: Neurexin, Wishful Thinking, Glass Bottom Boat, Futsch, Axon transport, Microtubule, BMP Signaling

***Address correspondence to:**
Swati Banerjee
Department of Cellular and Integrative Physiology
Long School of Medicine
University of Texas Health
7703 Floyd Curl Drive
San Antonio, TX 78229-3900, USA
email: banerjees@uthscsa.edu

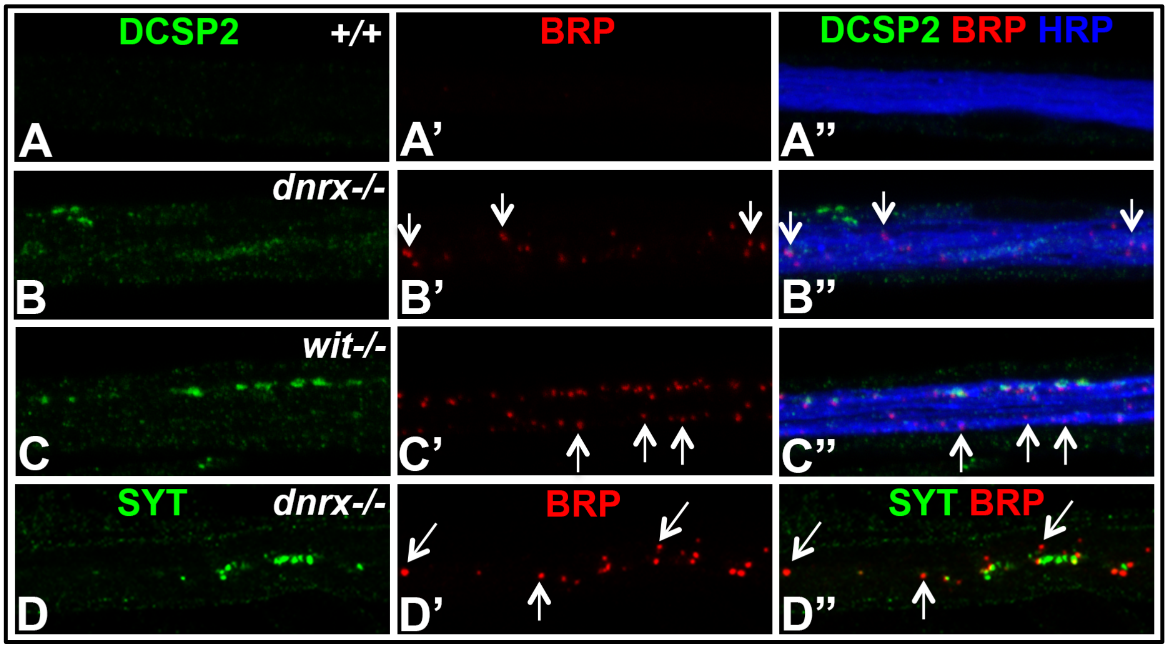

Supplemental Figure 1. Defect in axonal transport of synaptic cargoes in *dnrx* and *wit* mutants.

(A-C") Confocal images from a portion of larval segmental nerve from wild type (A-A"), *dnrx* (B-B") and *wit* (C-C") mutants triple labeled with antibodies against *Drosophila* Cysteine String Protein 2 (DCSP2, green, A-C), Bruchpilot (BRP, red, A'-C') and HRP (blue, A"-C"). *dnrx* (B-B") and *wit* (C-C") mutants show accumulation of DCSP2 and BRP punctae along the axons compared to wild type (A-A"). Many of the Brp-positive puncta do not overlap with the DCSP2 accumulation along the axons (white arrows, B', B" and C', C") in *dnrx* (B-B") and *wit* (C-C") mutants.

(D-D") Confocal images from a portion of larval segmental nerve from *dnrx* mutants labeled with anti-Syt (green, D, D") and anti-Brp (red, D', D"). White arrows (D',D'') indicate Brp positive puncta that do not associate with synaptic vesicle cargoes labeled with antibodies against Syt (green, D").

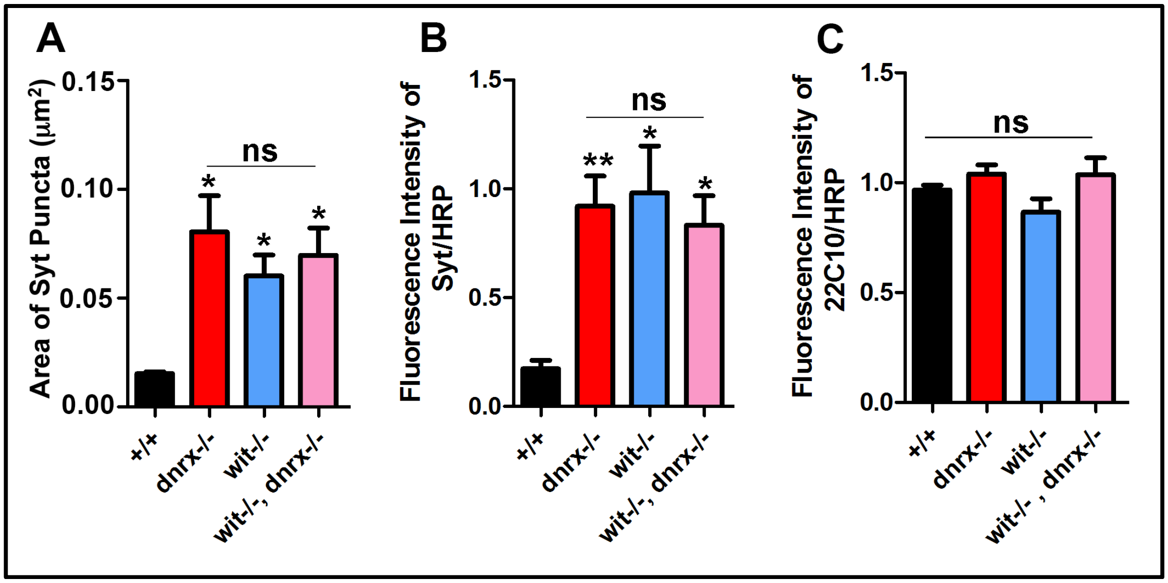

Supplemental Figure 2. Syt puncta area and intensity and Futsch intensity in single and double mutants of *dnrx* and *wit*.

(A-C) Measurements of Syt puncta area (A) and ratio of fluorescence intensity of Syt/Hrp (B) reveal a significant increase in segmental nerves of single and double mutants of *dnrx* and *wit* compared to wild type. Fluorescence Intensity ratio of Futsch (22C10)/Hrp (C) in segmental axons is similar across wild type, single and double mutants of *dnrx* and *wit.*

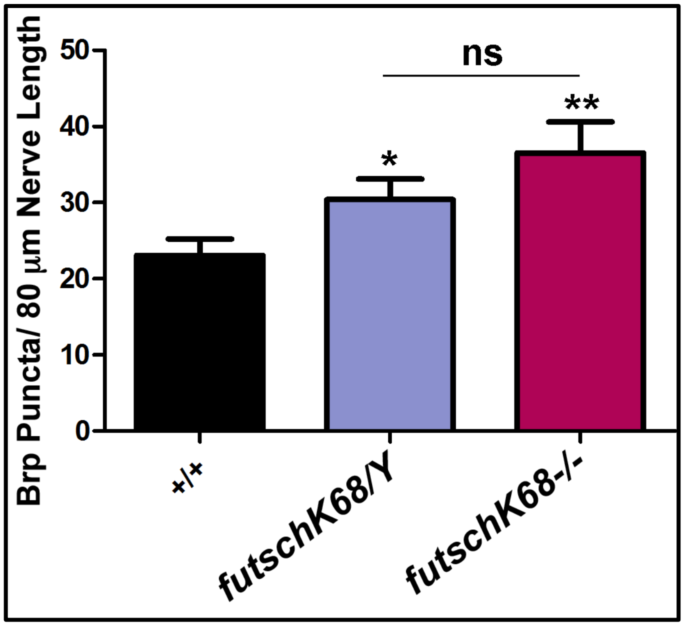

Supplemental Figure 3. Transport of active zone components labeled with Brp-positive puncta in *futsch* mutants.
futschK68/Y and *futschK68-/-* display significant increase in accumulation of Brp-positive puncta along distal segmental nerves compared to wild type.
n= 15 animals
Error bars represent mean $\pm$ SEM (***p<0.001, **p≤0.01, *p≤0.5, ns – not significant).

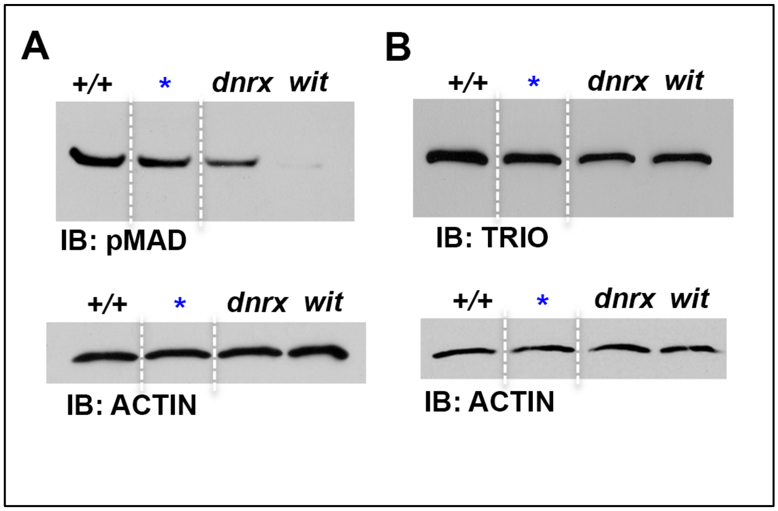

Supplemental Figure 4. (A, B) Scans of western blots, parts of which are represented in Fig. 6, show all lanes in the blots. The second lane characterized by the blue asterisk in all blots belongs to an unrelated genotype that is not reported in this paper. The dashed white lines depict the area that is removed from the gels shown in Fig. 6A, B.

Supplemental Table 1: Axon transport defects in various genotypic combinations used in this study

GENOTYPE	NORMAL TRANSPORT	DEFECT IN TRANSPORT	PARTIAL RESCUE OF TRANSPORT DEFECTS
+/+	✔		
dnrx/+	✔		
wit/+	✔		
dnrx-/-		✔	
dnrx/Df(3R)3C1		✔	
elav-Gal4; UAS-dnrx RNAi		✔	
OK6-Gal4/UAS-dnrx RNAi		✔	
elav-Gal4; UAS-dnrx		✔	
OK6-Gal4/UAS-dnrx		✔	
wit-/-		✔	
elav-Gal4; UAS-wit-GFP	✔		
elav-Gal4	✔		
wit/+; dnrx/+		✔	
wit/+; dnrx-/-		✔	
wit-/-; dnrx-/-		✔	
OK6-Gal4/UAS-dnrx; wit-/-		✔	
$futsch^{K68}$/Y		✔	
$futsch^{K68}$-/-		✔	
$futsch^{K68}$/+; dnrx/+		✔	
$futsch^{K68}$/Y; dnrx-/-		✔	
gbb-/-		✔	
mef2-Gal4/UAS-gbb	✔		
mef2-Gal4/UAS-gbb; dnrx/Df(3R)3C1			✔
elav-Gal4/UAS-gbb; dnrx/Df(3R)3C1		✔	
